# *BoCluSt*: Bootstrap Clustering Stability Algorithm for Community Detection

**DOI:** 10.1371/journal.pone.0156576

**Published:** 2016-06-03

**Authors:** Carlos Garcia

**Affiliations:** CIBUS Universidade de Santiago, Campus Sur, 15782 Santiago de Compostela, Galiza, Spain; University of Ulm, GERMANY

## Abstract

The identification of modules or communities in sets of related variables is a key step in the analysis and modeling of biological systems. Procedures for this identification are usually designed to allow fast analyses of very large datasets and may produce suboptimal results when these sets are of a small to moderate size. This article introduces *BoCluSt*, a new, somewhat more computationally intensive, community detection procedure that is based on combining a clustering algorithm with a measure of stability under bootstrap resampling. Both computer simulation and analyses of experimental data showed that *BoCluSt* can outperform current procedures in the identification of multiple modules in data sets with a moderate number of variables. In addition, the procedure provides users with a null distribution of results to evaluate the support for the existence of community structure in the data. *BoCluSt* takes individual measures for a set of variables as input, and may be a valuable and robust exploratory tool of network analysis, as it provides 1) an estimation of the best partition of variables into modules, 2) a measure of the support for the existence of modular structures, and 3) an overall description of the whole structure, which may reveal hierarchical modular situations, in which modules are composed of smaller sub-modules.

## Introduction

Complex systems are often modeled and analyzed as networks of related elements (nodes) connected by edges or links representing the relationship between them [1, 2]. In Biology, many of these networks show a modular structure: nodes can be grouped into communities or modules with a dense web of links among them, but with thin links between nodes in different modules [3–6]. This has been observed in gene expression networks [7, 8], protein-protein interactions [9], metabolic [10, 11] and developmental [12, 13] pathways, and also in species interactions in ecosystems [14, 15].

The building of models for these networks may require the use of module identification (also called community detection) procedures. Many such procedures have been proposed (see [16] for an ample review). Some are confirmatory, requiring a prior knowledge of module demarcations or at least of the number of modules present (e.g., [17, 18]). Other procedures ([19–21]) are exploratory and unsupervised, making no assumptions about modules and trying to identify the partitions among nodes that maximize some criterion of modular structure. Some of them consider heterogeneity in link weight, which may be fundamental for the understanding of the whole network [22]. By different means many try to maximize modularity, a measure of the density of links among nodes in the same module relative to that between nodes in different modules [23]:

$$Q=\frac{\text{1}}{\text{4}m}\underset{ij}{\sum }\left(A_{ij}-\frac{k_{i}k_{j}}{\text{2}m}\right)\delta \left(c_{i},c_{j}\right)$$

where *A*_*ij*_ = 1 if a link connects nodes *i* and *j* and *A*_*ij*_ = 0 otherwise, *k*_*i*_ is the degree of (number of links connected to) node *i*, *m* is the total number of links in the network, and the function δ yields 1 when modes *i* and *j* are in the same module and 0 otherwise. Furthermore, other procedures, called divisive, delete network links so that existing clusters get disconnected [24]; others are based on random walks, in which a walker moving randomly along the network edges is expected to make many repeated visits to variables in the same cluster, given the high density of edges among them [25]; others are based on statistical inference, i. e., they try to measure how well theoretical models of the network community structure fit the observed data [26], etc.

Given that many community detection procedures were designed to analyze very large networks in a reasonable time, they try to minimize the amount of resources required to perform their task, i. e., to minimize computational complexity. They also tend to work better when networks are sparse, i. e., when the overall link density is low, so that the number of links is similar to that of nodes [16]. However, more computing intensive methods can be used in the case of small to moderate sized data sets. Among these there are methods using stability as the criterion to validate module partitions [27, 28]. A clustering algorithm is applied to a series of data samples and the solution showing the highest replicability is taken as that corresponding to the true cluster structure. Some of the procedures based on this approach [29–31] generate the series of data samples by splitting the available data into random sub-samples, while others [28, 32] generate full-sized data samples by bootstraping. In contrast with most community detection procedures, which take between-node link information as a fixed input, stability based procedures use each generated sample to obtain information about these links, so that their results depend on the quality of the information on the links between nodes, or the estimation precision thereof. Of course, The robustness of module allocations may depend heavily on this quality [23]. This may not be critical when the aim of the analysis is the identification of large-scale community structures in big data sets, but could become so when the focus is on the allocation of particular variables to particular modules, as in the smaller networks considered in the analyses of gene regulation pathways and signal transduction cascades. In addition, stability procedures are robust in the sense that they use no stringent definitions of partition quality and they markedly reduce biases related to the spatial distribution of modules or the models about the data used by clustering algorithms [28]. However, these procedures can be demanding on computing resources, partly because they measure stability by making pairwise comparisons between module partitions obtained in different generated data samples [33], so the number of comparisons increases rapidly with the number of these samples.

This article introduces *BoCluSt*, a new stability based procedure for module identification in sets of correlated variables that is less computationally demanding than previous stability methods because it measures this stability by calculating the variance of a single vector of module coincidences instead of making multiple comparisons between generated samples. An input file containing individual observations for these variables is subjected to bootstrap resampling combined with a clustering algorithm, and the clustering solution showing the highest stability under resampling is selected. The procedure also uses resampling to generate a distribution of results under the null situation of no correlation between variables, which makes it possible to explicitly evaluate the support for the existence of community structure in the data. Computer simulation and the analysis of gene expression data show that, for data sets of a small to moderate size, *BoCluSt* tends to be superior to community detection procedures for which software is available, and that it can also detect the existence of hierarchical modular structures.

## Materials and Methods

### Implementation of *BoCluSt*

For a dataset of *n* variables *x*_*i*_ (*i* = 1, …, *n*), measured on *m* records, a clustering method is applied to obtain partitions into p clusters (2 ≤ *p* ≤ *n*-1); in the present implementation: k-means using the R *kmeans* function [34], an obvious choice, as it is the simplest and most popular clustering algorithm [35]; as an alternative using 1 –absolute Pearson correlations as distances between variables, there is also the option to use the *pam* function -Partition Around Medoids clustering algorithm- from the R package *cluster* [36]). Because of its use of the k-means algorithm, *BoCluSt* imposes no limits to link density in the data. Since all pairwise links between variables are considered, it works at maximum link density.

For a *p*-cluster partition, the “coincidence matrix” denoted by ***C***_***p***_ = [***C***_***p****ij*_] (*i*, *j* = 1, …, *n*) is defined as follows:

1. ***C***_***p****ij*_ = 1 if *x*_*i*_ and *x*_*j*_ are in the same cluster.
2. ***C***_***p****ij*_ = 0 if *x*_*i*_ and *x*_*j*_ are not in the same cluster.
3. by agreement ***C***_***p ii***_ = 1; *i* = 1, …, *n*.

Consequently, the matrix ***C***_***p***_ is symmetric, i. e. ***C***_***p****ij*_ = ***C***_***p****ji*_. The *(n*^*2*^*-n)/2* relevant, non-redundant (condition iii is irrelevant) elements in ***C***_***p***_ are stored in vector ***r***_***p***_, and the stability of each of the *n-2* cluster analyses is tested by bootstrap resampling of the individual observations in the original dataset to generate new full-size data sets and obtain new *n-2* vectors ^*B*^***r***_***p***_, where *B* denotes the ordinal number of bootstrap (*B* = 1, 2, 3, ….). For each bootstrap resampling *B*, the analysis of the distribution of the variables into *p* clusters gives us a new vector ^*B*^***r***_***p***_ and then the sequence of vectors {^*1*^***r***_***p***_, ^*2*^***r***_***p***_, …, ^*B*^***r***_***p***_, …}.

If a real, detectable module structure existed in the data, cluster analyses considering the appropriate number of clusters-modules would tend to allocate variables in the same clusters in all resamples, so that the variance across resamples would be low for each element in ^*B*^***r***_***p***_. A given pair of variables *x*_*i*_, *x*_*j*_ (*i ≠ j*) would tend to be either in the same cluster, the corresponding element value ^*B*^***r***_***p****ij*_ being equal to1 in most of resamples, i. e. ^*B*^***r***_***p****ij*_ = 1 for almost all *B*. However, if *x*_*i*_, *x*_*j*_ (*i ≠ j*) tend to be in different clusters, then ^*B*^***r***_***p****ij*_ = 0 in most of resamples. In analyses considering wrong numbers of clusters -or analyses of data with no community structure-, each bootstrap replicate would result in clusters containing random combinations of variables, and therefore in high variance of the elements of ^*B*^***r***_***p***_ across bootstraps. In *BoCluSt*, the variance of the vector corresponding to each *p*-partition, *Var*(^*B*^***r***_***p***_), is calculated as the sum of the variances of its elements across resamples. The partition resulting in the minimum sum of variances (i. e., the number of clusters *p* providing the most stable ^*B*^***r***_***p***_) would constitute the best estimate of the module structure in the original dataset.

However, the distribution of this sum of variances is not independent of the number of clusters considered in the successive *n-2* cluster analyses. To correct for this effect, the sums of variances are made relative to their expected values in a null situation with the same number of clusters and a lack of correlation between variables. This is obtained by randomizing the observed variable values independently across individuals, so that while univariate distributions are maintained, any correlation between variables disappears. The resulting null distribution for the sum of variances also makes it possible to evaluate the amount of evidence for modular structure in a particular data set. The corrected sum of variances (“variance criterion” in what follows) is used as an optimal partition criterion.

A summary of the steps taken is:

1. A series of bootstrap samples is taken from the original dataset of *m* records of *n* variables.
2. The clustering algorithm is applied to each sample to provide a set of *p* (from 2 to *n*-1) modules partitions.
3. In each B bootstrap sample and *p* value, a ^B^***r***_***p***_ vector of (*n*^*2*^*-n*)/2 relevant, non redundant variable cluster allocation coincidences is obtained. Then the variance of ***r***_***p***_ across bootstrap samples and the sum of variances in partition *p* are calculated.
4. The previous process is repeated for randomized, no-correlation samples of the original data set, to generate a distribution of the sum of variances for *p*-module partitions in the absence of any module structure. These averages of these distributions are used to normalize the sums obtained for the same *p* value in 5), and its percentiles to test for the existence of module structure for that partition.
5. The partition showing the lowest normalized sum of variances -variance criterion- is taken as the best clustering solution.

### Module identification

The performance of *BoCluSt* was studied in simulated datasets of grouped variables *x*_*ij*_:

$$x_{ij}=c_{i}+e_{ij}$$

where variable component *c*_*i*_ was common to all *x* variables in module *I*, causing correlation among these variables, and *e*_*ij*_ was specific to each *x*_*ij*_. The considered datasets are shown in Table 1.

**Table 1:** Cases considered in the computer simulations in Fig 1.

Case	Number of variables	Number of modules	Module sizes	Sample size	Components distribution	v(*c*)
a	8	2	4, 4	100	Normal	0.030
b	8	4	2, 2, 2, 2	100	Normal	0.030
c	8	2	4, 4	100	Normal	0.010
d	8	2	4, 4	100	Normal	0.015
e	8	2	4, 4	25	Normal	0.030
f	8	2	4, 4	50	Normal	0.030
g	8	7	1,1,1,1,1,1,2	100	Normal	0.030
h	8	3	5, 2, 1	100	Normal	0.030
i	8	2	4, 4	100	Beta	0.030*
j	8	2	4, 4	100	Uniform	0.030**
k	4	2	2, 2	100	Normal	0.030
l	16	2	8, 8	100	Normal	0.030

The cases differed in number of variables, number and sizes of modules (there were as many modules as sizes listed), number of individuals measured, distributions of the variables and variance of *c*, *v(c)*. In all cases, *e* had a variance of 0.050. The correlations corresponding to the three considered *v(c)* values were 0.375, 0.231 and 0.167.

* *c* was generated as a beta variable with parameters α = 0.246 and β = 2, and *e* as a beta variable with α = 0.625 and β = 2, using R function *rbeta*; the resulting *x*, *c* and *e* distributions were markedly asymmetric.

** *c* was generated as a uniform variable with the range 0 to 0.600 and *e* as a uniform variable with the range 0 to 0.775 using R function *runif*.

### Detection of hierarchical modular structures

The ability of *BoCluSt* to detect hierarchical modular structures (i. e., the presence of sub-modules within modules) was studied by simulating datasets of variables:

$$x_{ijk}=g_{i}+s_{ij}+e_{ijk}$$

where g_i_, s_ij_ and e_ijk_ were module, sub-module and variable-specific, respectively.

### Comparison with other procedures: computer simulation

Multi-sample simulations compared *BoCluSt* with the algorithm by Ahn et al [37] based on clustering the links between nodes instead of the nodes themselves and aimed at the detection of hierarchical and overlapping community structures, as implemented in the R CRAN package *linkcomm* [38]; with the standard *silhouette* cluster validation procedure [39], based on ratios of between-node distances, as implemented in the *cluster* R CRAN package; and with the community detection procedures in the *igraph* R CRAN package [40]. These were the *Edge betweenness* [41], a divisive method based on modularity; *Fastgreedy* [42], a hierarchical agglomeration algorithm based on modularity; *Infomap*, which finds the community structure that minimizes the expected description length of a random walker trajectory [43]; *Label propagation* [44], a nearly linear time algorithm that labels the network nodes and then updates these labels by majority voting in the neighborhood of the node; *Leading eigenvector* [45], based on calculating the leading non-negative eigenvector of the modularity matrix of a graph; the “*Louvain* procedure” [46], which finds community structure by optimizing modularity; *Optimal modularity* [47], which tries to maximize the modularity measure over all possible community partitions; *Spinglass* [48], based on simulated annealing, and *Walktrap* [49], which uses random walks on graphs to detect densely connected subgraphs, i. e., communities. These compared procedures took lists of weighted links between nodes-variables as input; here, the weights were the between-variables Pearson correlations calculated with the data generated in each simulation iteration.

### Comparison with other procedures: Analysis of gene expression data

These community detection procedures were also applied to the transcriptomic data in [50], corresponding to measures of nuclear gene expression in a *Drosophila melanogaster* experiment in which five different mitochondrial strains were introgressed in flies with an identical nuclear genetic background (NCBI accession GSE24729; CEL files, 40 samples; available at: http://www.ncbi.nlm.nih.gov/geo/query/acc.cgi?acc=GSE24729). Using *AmiGO 2* [51] and Bioconductor *affy* ([52]; RMA normalization method), *annaffy* [53], and *drosophila2*.*db* [54] libraries, the expression of two sets of genes were analyzed. First, of all, genes involved in cell growth proliferation, annotated with the ontologies GO:0030307, “positive regulation of cell growth” and GO:0030308, “negative regulation of cell growth”. It was possible to obtain expression measures for 24 probe sets corresponding to 22 genes in these ontologies. Secondly, the larger set of genes annotated as “DNA repair” (GO:0006281; 104 probesets in the data file, corresponding to 100 genes).

## Results and Discussion

**Fig 1:**
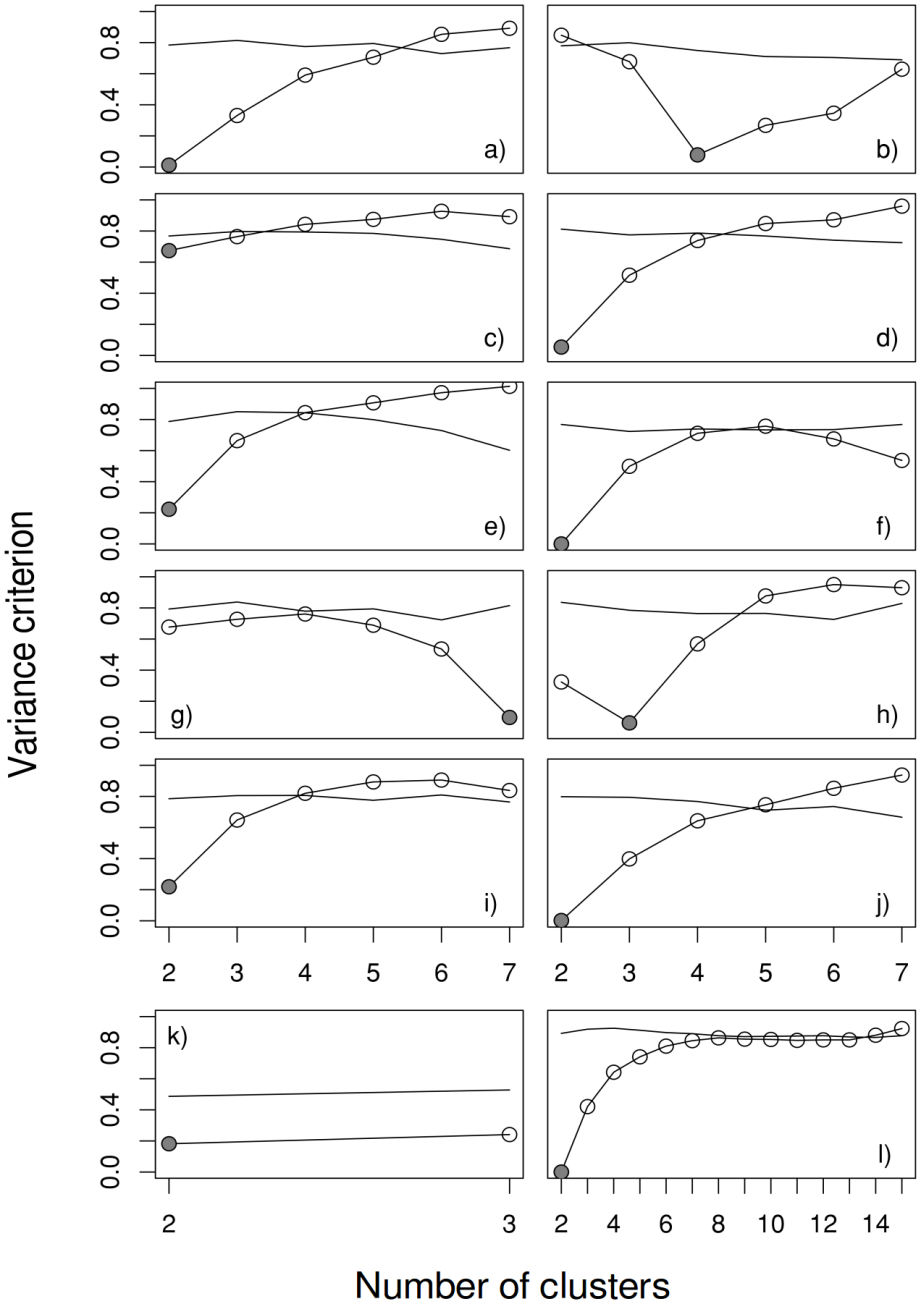
Values for the variance criterion (circles) in the cases listed in Table 1. A single, randomly taken simulated data set is shown per case, with 100 randomized null data sets and 500 bootstrap resamples per data set, along with the lower 2.5 percentile (simple lines) for the corresponding null situation of no correlation between variables. Grey circles mark the value for the true number of modules.

*BoCluSt* was able to identify the correct number of modules even for small-sized samples and also moderate correlations between variables in the same module (Fig 1). Thus, a sample size of 25 (Fig 1e) was enough to easily identify two modules of four variables with a within-module correlation of 0.375, and modules with within-module correlation of 0.231 were easily detected using samples with a size of 100 (Fig 1d). The performance of the procedure did not obviously depend on module number and size, the homogeneity of these sizes (Fig 1g and 1h) or the variables’ distributions (Fig 1i and 1j). The less favorable situations were those with the lowest correlation within modules (0.167, Fig 1c) and the lowest number of variables (four variables, Fig 1k). In the latter case, the variance criterion was clearly under the corresponding value for the null case, but the differences between the two and the three clusters solutions were very slight.

Fig 1 considers only from 2 to *n*-1 as the possible cluster numbers because it makes little sense to consider any coincidences in module allocation when there are *n* clusters of size one (and therefore no coincidences) or when there is a single cluster including all variables (total coincidence). However, because *BoCluSt* compares the obtained results with those expected under the absence of community structure, it is possible to conclude that there is no evidence for such structure when all the 2 to *n*-1 partitions are above the lower 2.5 percentile of the null distribution. Thus, *BoCluSt* does not only provide an estimate of the number of modules, but also of the reliability of that estimate and of the overall degree of modular structure in the data. It also makes it possible to compare the amount of evidence for alternative solutions.

**Fig 2:**
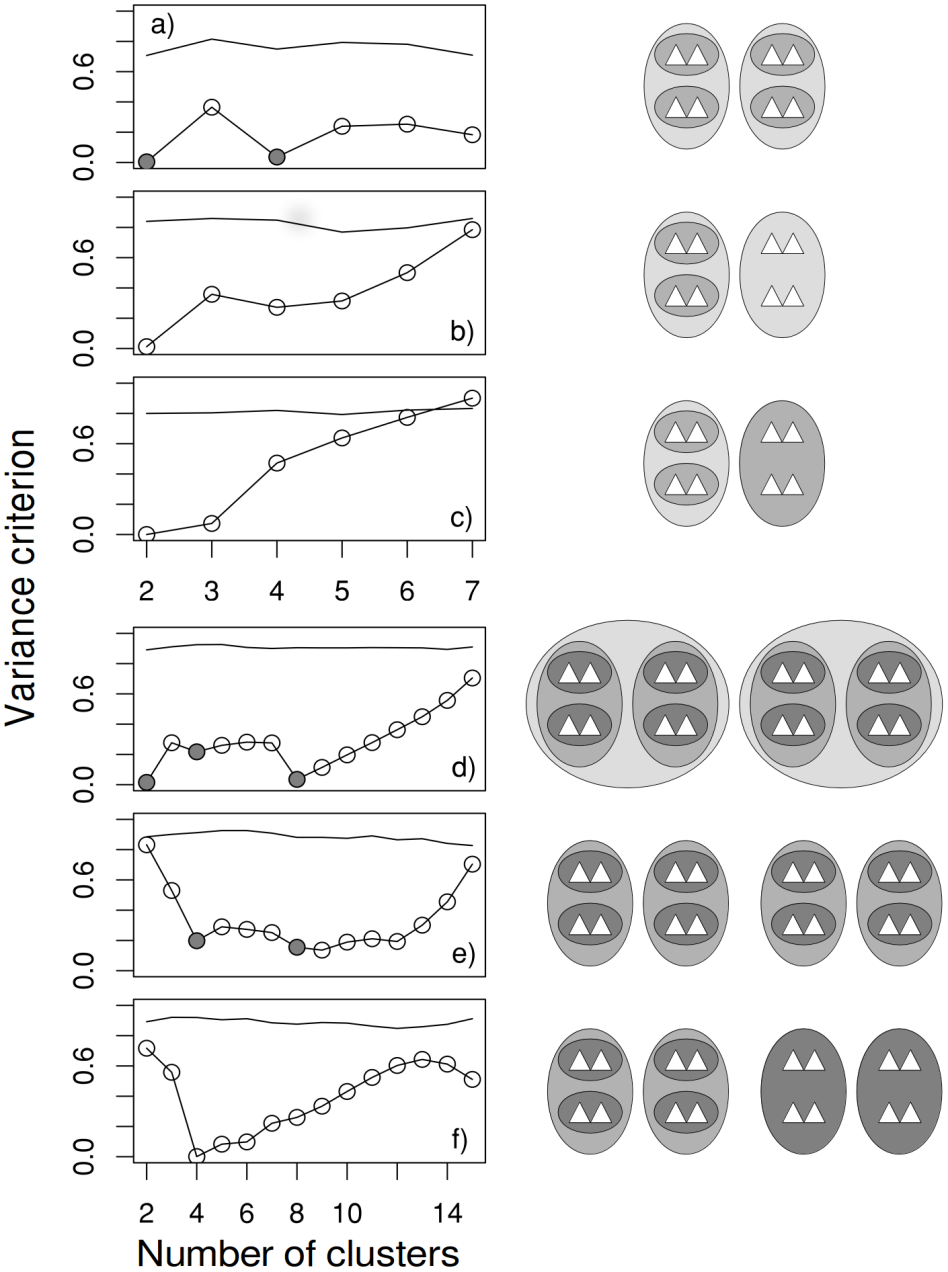
Analysis of hierarchical communities. A single, randomly taken simulated data set is shown per case, with 100 records, 100 randomized null data sets and 500 bootstrap resamples per data set. The graphs (left) show the variance criterion for all possible numbers of clusters along with the lower 2.5 percentile for the corresponding null situation of no correlation between variables (simple lines). Grey circles mark correct clustering results for regular partitions. The diagrams to the right represent the different situations. The grayscale indicates the value of the correlation between variables (triangles) in the same ellipse. These were 0.273 and 0.545 in the eight variable cases (a–c), and 0.214, 0.429 and 0.643 in the 16 variable cases (d to f).

*BoCluSt* was able to detect hierarchical modular structures, especially when the hierarchy was regular, i. e., the pattern of subdivision was the same in all modules (Fig 2a, 2d and 2e). These regular partitions appeared as local minima in the variance criterion profile: two and four modules in Fig 2a; two, four and eight modules in Fig 2d. The procedure failed in the case of four modules and eight sub-modules (Fig 2e), in which the second local minimum was found for nine clusters instead of eight. This suggests that the sample size required for module identification increases with module number. In any case, it must be noted that, while able to detect some hierarchical structures, *BoCluSt* does not provide a formal diagnostic for such structures.

Defining a single correct result became harder for less regular partitions. For example, in Fig 2b, both two or three module partitions would be possible. While the partition into two modules was easily detected, that into three modules resulted in a local maximum instead of a minimum. This maximum disappeared when the correlation between variables in the large module on the right of the diagram increased (Fig 2c), which, not unexpectedly, suggests that module detection is easier when within module links are strong. In any case, the low criterion values for two and three clusters seen in Fig 2c are not be clear evidence of hierarchical clustering, because the criterion values neighboring a minimum can also be low in non-hierarchical situations, as seen for example in Fig 1h and 1k, or in the case of partially overlapping modules.

Fig 2e shows many consecutive low values for the variance criterion. This could be related to the fact that many partitions (into four, five, six or eight modules) are possible in that case. However, this could not explain all results. The criterion values remained low beyond eight, the last “correct” number of clusters. In any case, the comparison of Figs 1 and 2 suggests that profiles showing more than one point of inflexion may be indicators of hierarchical modular structures.

**Fig 3:**
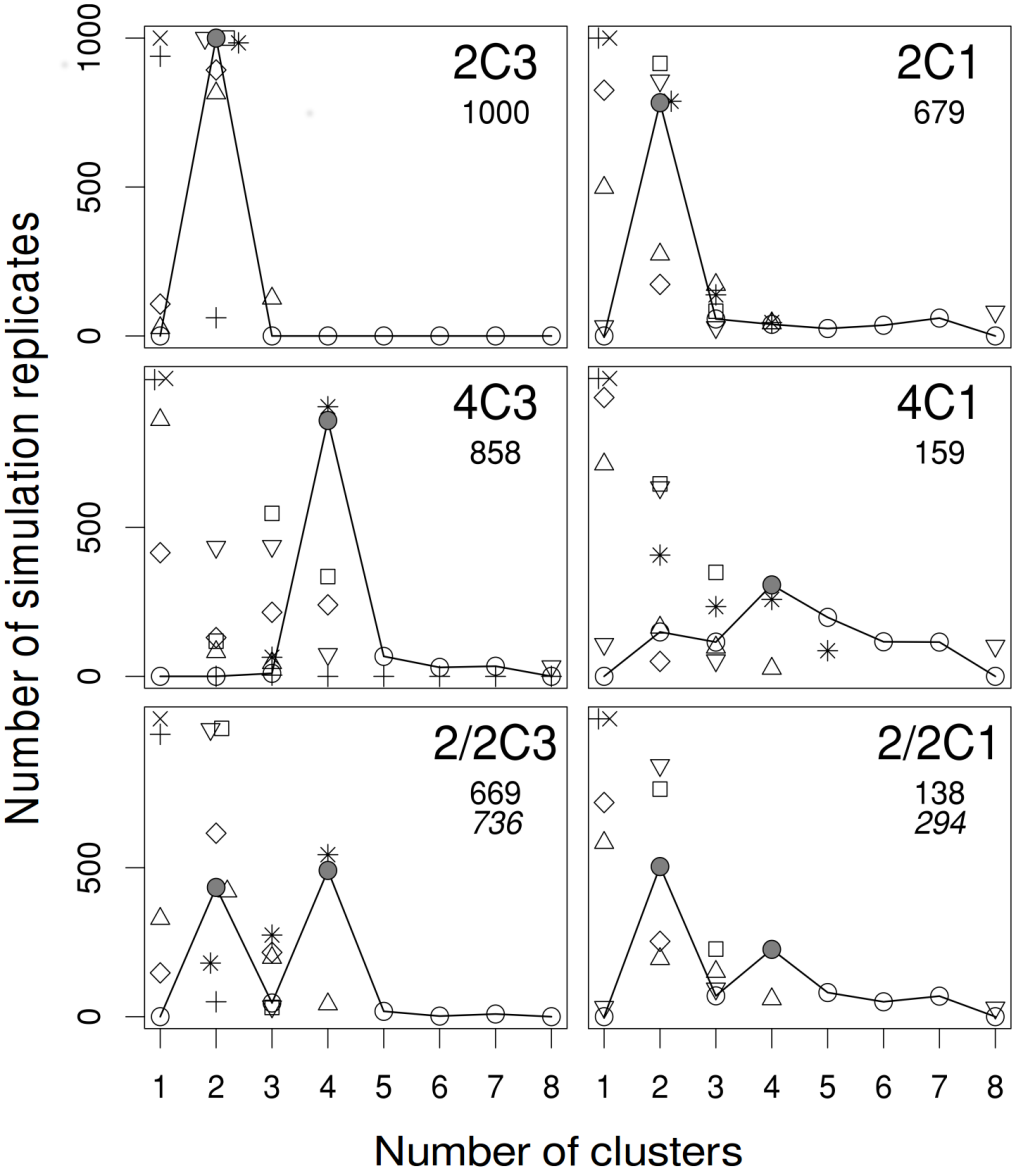
Frequency of numbers of modules detected in computer simulations comparing the different procedures. There were eight variables, 100 records and 1000 replicates per case. 2C and 4C are cases with two and four variable modules respectively, and 2/2C, hierarchical situations of two modules each divided in two sub-modules. The numbers to the right of the Cs mark the variance of component *c*, common to variables in the same module or sub-module: 1, variance = 0.01; 3, variance = 0.03 (the variance of component *e* was = 0.05 in all cases). Circles, upwards triangles, plus signs, x signs, rhombs, squares, asterisks, and downwards triangles show results for the *BoCluSt*, *Edge betweeness*, *Infomap*, *Label propagation*, *linkcomm*, *Louvain*, *silhouette*, and *Walktrap* procedures, respectively. In these simulations, the results for *Fastgreedy*, *Leading eigenvector*, *Optimal modularity* and *Spinglass* procedures were very similar to those of *Louvain*; only the latter is shown for clarity. Also for clarity, counts lower than 25 are shown for *BoCluSt* only. Grey symbols mark the true number(s) of modules. The smaller font values are the numbers of replicates in which *BoCluSt* found results both correct and significant (under the 2.5 quantile of the null distribution). In the hierarchical cases, these results consisted in two significant minima in the variance criterion for two and four clusters. In italics, the number of replicates finding the same two minima, whether significant or not.

In the computer simulation comparisons between procedures, the *BoCluSt*, *silhouette*, *Louvain* and *Walktrap* procedures never failed to identify two modules for sample sizes of one hundred and moderate within module correlations of 0.375 (Fig 3 2C3). *Louvain* and *Walktrap* were better than *BoCluSt* when the correlation was reduced to 0.167 (Fig 3 2C1). However, all procedures except *silhouette* were clearly worse than *BoCluSt* in the case of four modules, as they failed to find four modules as the most frequent result when the correlation was 0.375 (Fig 3 4C3) and completely failed to detect them when the correlation was 0.167 (Fig 3 4C1). In the same situations, the right solution of four modules was that most frequently found by *BoCluSt*. The *silhouette* procedure slightly surpassed *BoClust* in performance in the 4C3 case but tended to underestimate the number of clusters when the correlations within clusters decreased in the 4C1 case.

In the hierarchical cases, most procedures tended to find only one or two modules in most replicates. The *silhouette* procedure quite frequently obtained an intermediate wrong result of three modules, whereas *BoCluSt* found two and four modules as the most frequent solutions. It was able to detect the hierarchical structure in most replicates when the correlation was moderate (Fig 3 2/2C3, in italics), but only in a minority when the correlation was low (Fig 3 2/2C1). The *Edge betweenness* and *Label propagation* procedures did particularly badly in the small number of variables cases considered in these simulations. This was also the case for *Infomap* and *linkcomm*, which failed to find any module structure in almost all simulation replicates. This was due to the maximum link density in these data sets. The removal of low-weight (i. e., low correlation) links improved markedly both procedures' ability to detect modules (not shown). However, in a practical situation this filtering would require to set an arbitrary threshold for link weights, which would limit the procedures' utility for unsupervised module detection.

*BoCluSt* outperformed the other procedures in most simulations made here, especially in small module cases. The low performance of some of these procedures was likely related to the fact that they are based on measuring modularity. Modularity-based methods face a “resolution limit in community detection”, which is most likely to occur when the number of module internal links is of the order of the square root of twice the total number of links in the network or smaller [55], i. e., when modules are small. *BoCluSt* seems to be unaffected by that limit, since it can easily detect two-variables modules (see Fig 1b). However, the use of bootstrap resampling by this procedure might command too many computational resources to be practical for the analysis of data sets of many variables, as those in genome-wide or human social networks. Thus, *BoCluSt* could be an alternative to low computational complexity, large-scale procedures in the analysis of moderate-sized data sets. The compared procedures would not be equivalent to *BoCluSt* for any problem size, however, because they do not use the same kind of information. Instead of starting with a previously known set of link weights, *BoCluSt* simultaneously estimates both weights and community structure.

**Fig 4:**
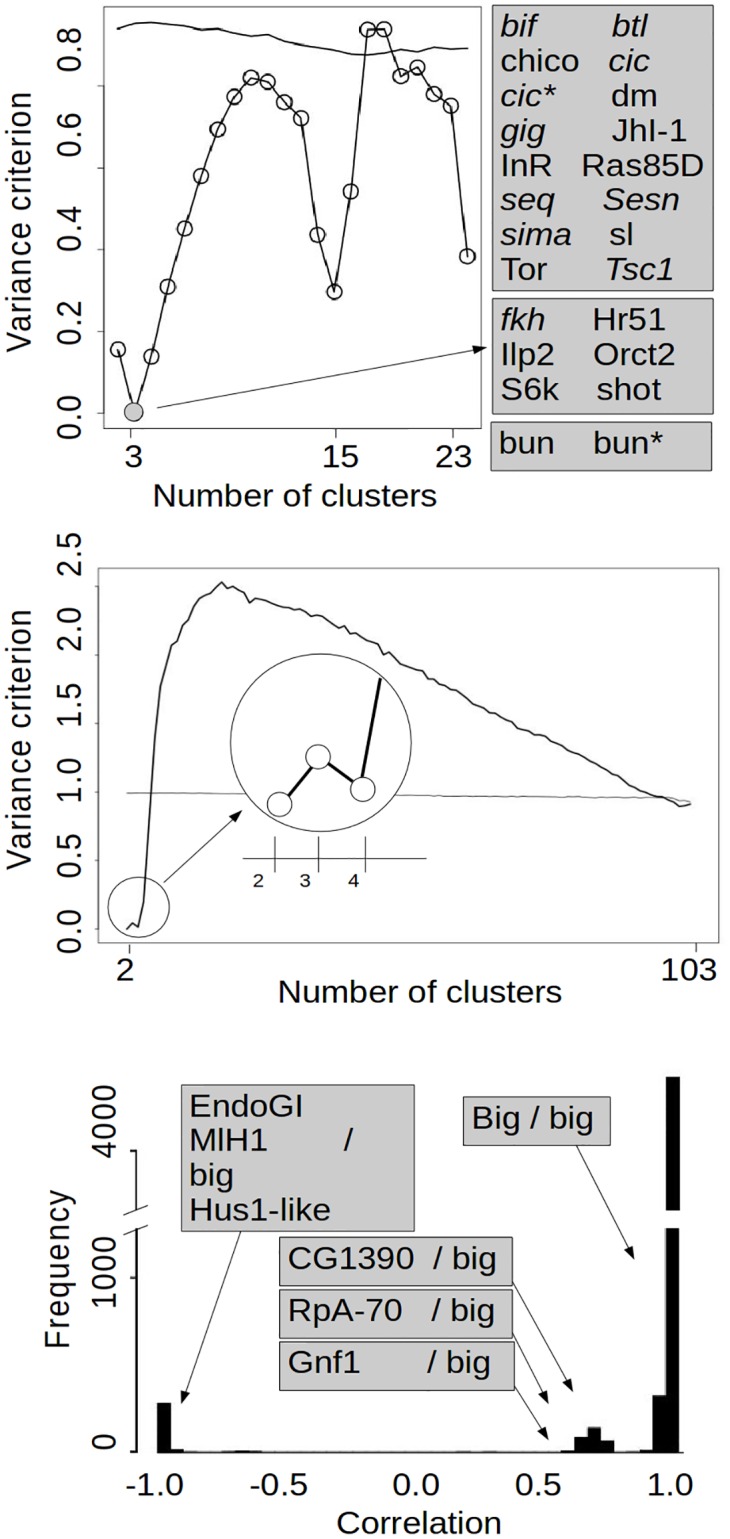
*BoCluSt* analysis of *Drosophila melanogaster* microarray data. Top, analysis of probes for 23 genes annotated in the ontologies “positive control of cell growth” and “negative control of cell growth” (in normal and italic fonts respetively). The gene composition of the three-clusters partition with the least value for the variance criterion is shown in the grey boxes to the right of the graph. Asterisks mark second probes of genes having two different probes in the array. Middle, analysis of 104 probe sets of genes annotated in the ontology “DNA repair”. The partition showing the lowest variance criterion is amplified in the circle. Bottom, frequencies of pairwise correlation values between the probe sets corresponding to DNA repair genes (5356 correlations among 104 probe sets). The analysis identified a big cluster of 98 genes of narrowly correlated expressions. The bars correspond to the correlations a) of these 98 probe sets among themselves (”Big/big” box); b) of these probe sets with the *EndoGl*, *Mlh1* and *Hus1-like* probe sets (left box); and c) with the *CG1390*, *RpA-70* and *Gnf1* probe sets (the three boxes at center; see the text for more detailed explanations).

In the analysis of the expression of genes involved in cell growth regulation (Fig 4 up; k-means analysis; 100 bootstrap replicates for both the observed and the randomized null cases; 100 such null cases were done), *BoCluSt* found clear evidence for three modules (the variance criterion for this solution was zero, showing that variables were placed in the same three clusters in all bootstrap resamples), and also for a hierarchical modular structure, as the results suggested that the three modules were composed of 15 and 23 sub-modules.

Most compared procedures tended to detect only two groups, i. e., they did not detect a third cluster including the two probe sets corresponding to gene *bun* (Table 2). Only *Walktrap* found the same structure as *BoCluSt*.

**Table 2:** Number and sizes of clusters found by the compared procedures in the analysis of gene expression.

Procedure	Cell growth		DNA repair	
	# Clusters	Sizes	# Clusters	Sizes/Composition
*BoCluSt*	3	16, 6, 2	2	(Big), (c, C_1_, q)
*Edge betweeness*	8	17, 1, 1, 1, 1, 1, 1, 1	4	(Big), (c), (C_1_), (q)
*Fastgreedy*	2	16, 8	1	-
*Infomap*	1	24	1	-
*Label propagation*	1	24	1	-
*Leading eigenvector*	2	16, 8	2	(Big), (A, B, c, C_1_, C_2_, p, q)
*Linkcomm*	1	24	1	-
*Louvain*	2	16, 8	2	(Big), (A, c, C_1_, q)
*Optimal modularity*	2	16, 8	2	(Big), (B, c, C_1_, q)
*silhouette*	2	15, 9	2	(Big), (T, R)
*Spinglass*	2	16, 8	2	(Big), (B, c, C_1_, q)
*Walktrap*	3	16, 6, 2	2	(Big), (c, C_1_, q)

The simpler partitions found for genes in the “DNA repair” ontology made it possible to list the genes in the smaller clusters (A, *Arp5*; B, *Blm*; C_1_, *CG10694*; C_2_, *CG18004*; c, *cry*; p, *p53*; q, *qjt*; R, *RpLP0*; T, *Tctp*; Big, all genes in the data set but those listed for each procedure). Genes within the same parenthesis set were in the same cluster.

In the case of the DNA repair genes, *BoCluSt* found a global minimum variance criterion for two clusters, and a local minimum for four clusters (Fig 4, middle). The correlation structure for the expressions of these probes was remarkably simple (Fig 4, bottom), which makes it easier to discuss the clustering results. There were 98 narrowly and positively correlated (r > 0.8) probe sets, three probe sets (corresponding to genes *CG10694*, *cry* and *qjt*) tightly correlated (r > 0.96) among themselves and with strong negative correlations (r < -0.88) with the previous 98, and finally three probe sets corresponding to genes *Blm*, *CG18004* and *p53* with positive correlations (from 0.55 to 0.73) with the big group of 98, negative correlations with the previous set of three (from -0.73 to -0.62), and moderate ones among themselves (0.19 to 0.61). Notice that the visible bars in the histogram in Fig 4 correspond to the numerous correlations involving the big group of 98 probe sets; the correlations among the other 6 probe sets were too few for visible bars. Thus, three “bar islands”can be seen in the histogram: on the left, the correlations of *CG10694*, *cry* and *qjt* with the variables in the big group, in the center, the correlations of *Blm*, *CG18004* and *p53* with the big group, and on the right, the correlations within the big group.

The *BoCluSt* results describe this structure. The solution with the largest support (two clusters) separates *cry*, *CG10694* and *qjt*, narrowly correlated among themselves and negatively correlated with the rest. In the also well supported solution of four clusters, *CG18004* and *p53* are separated from the larger group to constitute two single-variable clusters, which is not unexpected given that the two genes are not narrowly correlated to each other. *Blm* separates last, again in a single variable (fifth) cluster. It is not obvious, however, why the variance criterion increased markedly for this five-clusters solution (Fig 4, middle), given that the correlations for *Blm* were similar to those of the previous two.

In any case, *BoCluSt* provided a clearly better description of the community structure among these 104 probe sets than the compared procedures (Table 2), which, for example, included genes *cry*, *CG10694* and *qjt* and the negatively correlated *Blm* in the same cluster (*Louvain* procedure); allocated these three first genes in different clusters, despite the extreme positive correlations among them (*Edge betweenness*); or included *Arp5*, which had a correlation profile typical of genes in the big group of correlated genes, in a second cluster of seven genes (*Leading eigenvector*). Only *Walktrap* found the same solution as *BoCluSt*, as it had done for the smaller analysis of Cell Growth genes. However, *BoCluSt* and *Walktrap* are not equivalent. First, as shown by the computer simulations, *Walktrap* tended to fail as the number of communities increased (Fig 3); second, it does not take into account the precision in the calculation of link weights, and third, it could not detect hierarchical communities (*igraph* provides the modularity measures corresponding to all cluster partitionings of the variables -not shown-, and no clear local minima is seen in this series of measurementss for any of the microarray data sets, in contrast with what can be seen in Fig 4 for *BoCluSt*). The *silhouette* procedure did not reflect the correlation structure discussed above. The *RpLP0* and *Tctp* genes constituting the second cluster identified by this procedure had expressions which were very similar (correlations ~ 0.97) to those of genes allocated to the Big group by the remaining procedures, and high and negative correlations (< -0.93) with genes *CG10694*, *cry* and *qjt*. These poor results were to some extent unexpected, as *silhouette* works best in situations with compact, clearly separated and roughly spherical clusters [39], as was apparently the case with these DNA repair genes data. One possibility is that the performance of *silhouette* was impaired by the very large differences in module size. As already observed for cell growth regulation genes, *Infomap* and *linkcomm* (and also *Label propagation*) were unable to detect any module structure. This could be related to link density, which was rather extreme in this data set, including 98 narrowly correlated variables. As mentioned above, the performance of these two procedures in the computer simulations was much better when link density was artificially reduced. This highlights the fact that the comparisons made in this article had to use the particular data structure required by *BoCluSt*, which could partly explain the advantage found for this procedure. *BoCluSt* uses a file of individual observations for some variables as input and then estimates the weights of all possible links between these variables. Thus, it is designed to work at maximum link density, whereas the alternative procedures were designed to use a limited list of links and weights from somewhat sparse networks. Therefore, it is possible that these procedures do not work at their best when applied to the full density link sets specified by correlation matrices, as done here. However, this full-density, exploratory community detection considering all possible links between variables may be appropriate in a wide range of situations, such as the gene expression studies considered here.

## Conclusions

*BoCluSt* may be a valuable and robust tool in community detection analysis, as it provides 1) an unsupervised estimation of the number of modules and their composition; 2) a measure of the amount of evidence for this estimation and alternative module partitions, and 3) an overall description of the modular structure of the whole data set, which may reveal the existence of hierarchies of modules and nested sub-modules. Because of its unsupervised nature, *BoCluSt* can be used in automated analyses or in comparisons between data sets. The procedure uses k-means or pam as clustering algorithms in its present implementation, but its method of evaluating alternative partitions based on measuring their stability under resampling, in principle, would be compatible with any clustering algorithm. While permitting a more detailed analysis of community structure and the evaluation of alternative module partitions, its increased computational complexity would restrict its use to small to moderate data sets. However, this article shows that it tends to outperform alternative procedures for these kinds of data sets when network density is high. *BoCluSt* code is written in R and available at http://sourceforge.net/projects/boclust/files/BoCluSt.txt/download It requires the use of the *pam* function from *cluster* R package in case the Partition About Medoids clustering algorithm is chosen.
